# Spatial variation and associated factors of home delivery among reproductive age group women in Ethiopia, evidence from Performance Monitoring for Action Ethiopia Survey 2019, spatial and multilevel logistic regression analysis

**DOI:** 10.3389/fgwh.2024.1474762

**Published:** 2024-12-16

**Authors:** Ermias Bekele Enyew, Kokeb Ayele, Lakew Asmare, Fekade Demeke Bayou, Mastewal Arefaynie, Yawkal Tsega, Abel Endawkie, Shimelis Derso Kebede, Abiyu Abadi Tareke, Kaleab Mesfine Abera, Natnael Kebede, Mahider Shimelis Feyisa, Mengistu Mera Mihiretu

**Affiliations:** ^1^ Department of Health Informatics, School of Public Health, College of Medicine and Health Sciences, Wollo University, Dessie, Ethiopia; ^2^ Department of Health Promotion, School of Public Health, College of Medicine and Health Sciences, Wollo University, Dessie, Ethiopia; ^3^ Department of Epidemiology and Biostatistics, School of Public Health, College of Medicine and Health Sciences, Wollo University, Dessie, Ethiopia; ^4^ Department of Reproductive and Family Health, School of Public Health, Wollo University, Dessie, Ethiopia; ^5^ Department of Health System and Management, School of Public Health, College of Medicine and Health Sciences, Wollo University, Dessie, Ethiopia; ^6^ Amref Health Africa in Ethiopia, West Gondar Zonal Health Department, Gondar, Ethiopia; ^7^ Department of Medical Laboratory, College of Health Science, Debre Tabor University, Debre Tabor, Ethiopia

**Keywords:** home delivery, spatial variation, reproductive age women, Performance Monitoring for Action, PMAS, Ethiopia

## Abstract

**Introduction:**

Home birth is described as a delivery that takes place at home without the presence of a skilled birth attendant. In 2017, nearly 295,000 mothers died from various pregnancy and childbirth-related problems, accounting for approximately 810 maternal deaths per day. Therefore, this study aims to investigate the spatial distributions of home birth and associated factors in Ethiopia using the Performance Monitoring for Action Survey (PMAS) 2019) to get information that helps to take geographic-based interventions and can assist health planners and policymakers in developing particular measures to reduce home deliveries.

**Method:**

In PMA-ET 2019, a community-based cross-sectional study was conducted in collaboration with Addis Ababa University, Johns Hopkins University, and the Federal Ministry of Health from September 2019 to December 2019, in Ethiopia. A multi-stage cluster sampling procedure was employed to draw from the stratified 2019 PMAS sample. A weighted total of 5,796 women were included in this study. ArcGIS version 10.7 software was used to visualize the spatial analysis. In addition, STATA version 14 of the statistical software was used for multilevel analysis The Bernoulli model was applied using Kulldorff's SaTScan version 9.6 software to identify significant purely spatial clusters for home delivery in Ethiopia. Intra-class Correlation Coefficient (ICC), Likelihood Ratio (LR) test, Median Odds Ratio (MOR), and deviance (−2LLR) values were used for model comparison and fitness. Adjusted Odds Ratios (AOR) with a 95% Confidence Interval (CI) and *p*-value <0.05 in the multilevel logistic model were used to declare significant factors associated with home delivery.

**Result:**

The spatial distribution of home delivery was non-random in Ethiopia. Statistically significant high hotspots of home delivery were found in Somali, Afar, Sidama, most of South Nation Nationality and People Region (SNNP), most parts of Amhara, south west Ethiopia, and Oromia region. In the multilevel logistic regression model; Women from the lowest wealth quintile were 1.68 times [AOR = 1.68; 95% CI: 1.31, 2.15] higher odds of giving birth at home as compared to their counterparts. Regarding maternal educational status, mothers who had no education, primary education, and secondary education had 9.91 times [AOR = 9.91, 95% CI: 5.44, 18.04], 6.62 times [AOR = 6.62, 95% CI: 3.65, 12.00] and 2.99 times [AOR = 2.99, 95% CI: 1.59, 5.63] higher odds of giving birth at home compared to mothers who attained higher education, respectively. In addition, community-level factors were significantly associated with home delivery, women who had high community-level poverty were 1.76 times [AOR = 1.76; 95% CI: 1.14, 2.72] higher odds of home delivery compared to women who had low community-level poverty.

**Conclusion:**

Home delivery was statistically found to be a significantly high hot spot in Somalia, Afar, Sidama, most of the South Nation Nationality and People area (SNNP), most of Amhara, southwest Ethiopia, and the Oromia region of Ethiopia. Significant factors associated with home delivery in Ethiopia were women with lower levels of education, poor wealth, living in rural areas, high levels of community poverty, divorced or separated widowed marital status, and older maternal ages. Therefore, health institutions, health professionals, National and regional policymakers health planners community leaders and all concerned should give priority to the identified hot spot clusters to design an effective intervention program to reduce home delivery.

## Background

Home birth is described as a delivery that takes place at home without the presence of a skilled birth attendant (midwife, nurse, or doctor) (1). Maternal health is a top concern in the global health agenda (2). Though the majority of maternal deaths are preventable, women lose their lives due to complications related to pregnancy and childbirth every minute of every day, somewhere in the world (3). In 2017, nearly 295,000 mothers died from various pregnancy and childbirth-related problems, accounting for approximately 810 maternal deaths per day in the world (3). Except for a few countries (Benin, Namibia, Zimbabwe, and Vietnam), the use of professional care during delivery is significantly lower in Sub-Saharan Africa and South/Southeast Asia, according to findings from levels and trends in the use of maternal health services in developing nations (4). In Ethiopia, the percentage of women who gave birth at home was highest in the Afar and Somali regions (89.6 percent and 81.7 percent, respectively), while just 3.3 percent of Addis Ababa residents gave birth at home (4). Reducing the proportion of home births is a key strategy for lowering the maternal death rate (3).

Complications associated with pregnancy and childbirth account for considerable pregnancy and childbirth-related fatalities and disabilities worldwide, particularly in underdeveloped nations (5). Prolonged/obstructed labor, complications from a botched abortion, bleeding, malaria during pregnancy, anemia, and sepsis are the leading causes of death (5). Obstetric complications such as maternal morbidity and mortality are a result of home birth (6). The burden of home delivery, particularly unattended delivery, is not simply a mother health issue; it also results in perinatal and neonatal illness and mortality (7). In-home deliveries were found to have a 21% higher perinatal mortality rate than institutional deliveries (8). On the other hand, home deliveries are linked to infection and other negative neonatal and maternal outcomes (7).

Several studies evidenced that, educational status of women (7, 9–14), cultural factors (10, 11, 14, 15), region (9), parity (9), limited access to health facilities (10, 11), poor quality of care (10, 11), lack of transportation (10, 11, 16), age (4, 12, 13, 15, 17), marital status (16), environment (4, 18), distance to the health facility (3, 7, 9, 11, 16, 19), source of information (3, 7, 9, 16), antenatal care visit (3, 9–11, 20), birth order (9, 10), wealth index (9, 10, 12, 21), place of residence (3, 4, 9, 11, 13, 20), religion (9, 10, 15),employment of women and husband (4), knowledge on place of delivery (3, 12, 13, 20), Unplanned pregnancy (7) and decision on place of delivery (3, 12, 14) were statistically significant predictors of home delivery.

The existing literature on home deliveries in Ethiopia has primarily utilized the Ethiopian Demographic and Health Survey (EDHS) dataset (9), but a spatial analysis of the more recent PMA-ET 2019 data is lacking. This study aims to fill this gap by examining the spatial variation of home deliveries across Ethiopia and identifying the individual and community-level factors associated with this outcome. Using advanced spatial modeling techniques, the researchers will uncover the geographic patterns and clustering of home births, providing valuable insights into the regional differences. Furthermore, the study will contrast these findings with previous research, highlighting any changes over time and the implications for targeted maternal health interventions. By leveraging the newer PMA-ET 2019 dataset, this study promises to offer a comprehensive understanding of the spatial determinants of home deliveries in Ethiopia, ultimately informing more effective, evidence-based policies and programs to improve maternal and child health outcomes. Therefore, this study aims to investigate the spatial distributions of home birth and associated factors in Ethiopia using the Performance Monitoring for Action Survey (PMAS) 2019.

### Conceptual framework

**Figure 1:**
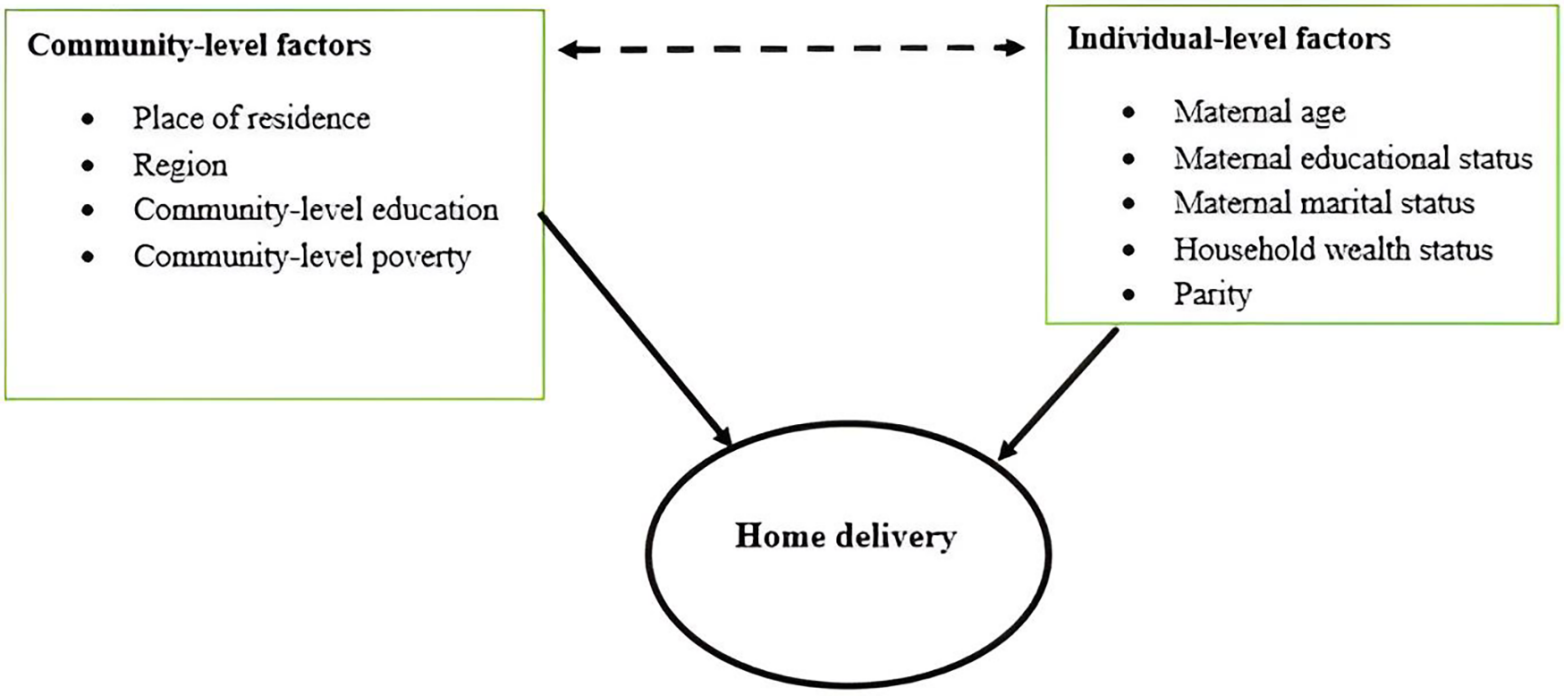
Conceptual framework for home delivery among reproductive age women. Adopted from (3, 4, 12, 13, 20).

This conceptual framework is composed of different literature and includes both individual-level and community-level factors (Figure 1) that affect spatial variation home delivery among reproductive age group women.

## Methods

### Study design and study period

The PMA Ethiopia 2019 Cross-sectional Household and Female survey generated secondary data in the study. The period of this cross-sectional study was September 2019 to December 2019 (22).

### Study area

**Figure 2:**
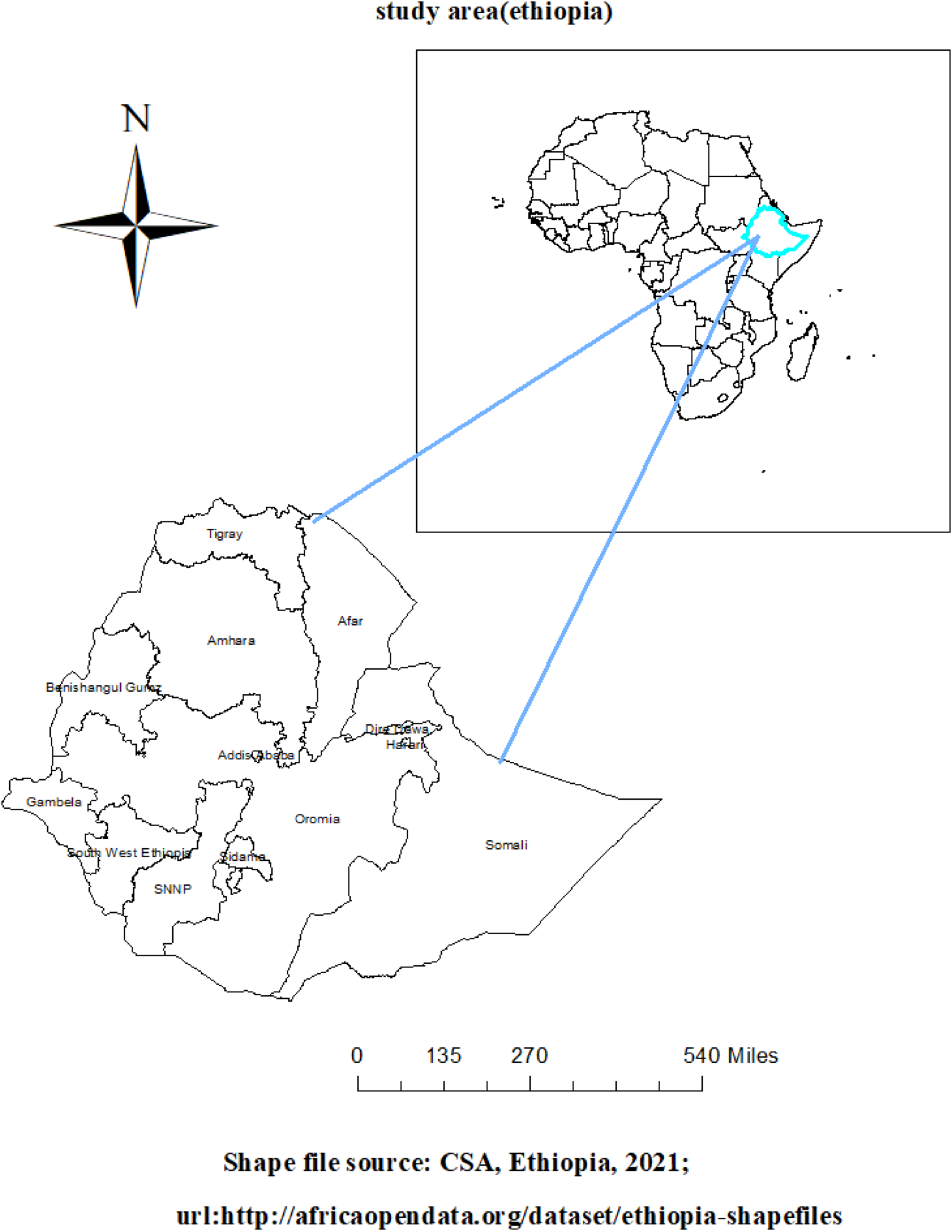
Map of study area (Ethiopia).

Ethiopia was the site of the investigation. The Horn of Africa is where Ethiopia is situated. Situated between 3o and 48°E, and 3o and 14oN on the African continent, Ethiopia is the second most populous country. Ethiopia has 133,352,010 people living there as of 2024; according to World Meter's elaboration of the most recent United Nations data, 77.9% of them reside in rural regions (23). It consists of two city administrative areas (Addis Ababa and Dire-Dawa) and eleven regional states [Tigray, Afar, Amhara, Benishangul-Gumuz, Gambella, Harari, Oromia, Somali, southwest Ethiopia, Sidama, and Southern Nations, Nationalities, and People's Region (SNNP)] (Figure 2).

### Study and source of population

Women who had given birth within the five years before each recent birth survey comprised the study population. All Ethiopian women in the reproductive age range (15–49) formed the source population.

### Sampling technique and sample size

A multi-stage cluster sampling procedure was employed to draw a probability sample of households and women of reproductive age from the stratified 2019 PMA-ET sample. Using a screening question form, all pregnant or immediately postpartum women residing in the EAs were identified as eligible participants in the panel survey. Altogether, 265 Enumeration Areas (EAs), or geographic sampling units, were drawn separately from rural and urban strata within the Tigray, Amhara, Oromia, and South Nation, Nationalities, and Peoples (SNNP), whereas randomly selected with probability proportional to size within Afar region without rural or urban stratification. All EAs were drawn from urban areas without stratification since Addis Ababa is exclusively urban. All women aged 15–49 who were either permanent residents of the selected households or visitors who slept in the household the night before the survey were eligible to be interviewed. A weighted 5,796 reproductive age group women who had a complete answer to all variables of interest from the total of 8,837 women participants in PMA-ET 2019 were selected.

### Variable of study

#### Outcome variable

The outcome variable taken as a binary response woman gave birth at home and others home coded as “1” which is home delivery, and women gave birth to different governmental health facilities, private health facilities, and non-governmental health facilities coded as “0” which is health facility delivery.

#### Independent variable

The independent variables were residence, maternal age, women's educational status, wealth status, parity, marital status, region, community-level poverty, and literacy.

#### Operational definition

##### Community level poverty

Number of female household members from the lowest and poorest wealth index categories. High poverty levels were defined as those who fall at or above the median value of the variables, whereas low poverty levels are defined as those who fall below the median value of the variables. Since the normalcy test of community-level poverty is skewed (the Jarque-Bera test's *p*-value was less than 0.05, indicating a skewed distribution), the median is utilized as the cut point in this case.

##### Community level literacy

percentage of mothers and other caregivers who have completed primary school and higher. We classified this literacy at the community level in a manner that was comparable to that of poverty.

### Data collection producer

Three separate research activities comprise PMA-Ethiopia, a five-year (2019–2023) project executed in collaboration with Addis Ababa University, Johns Hopkins University, and the Federal Ministry of Health. These include yearly cross-sectional surveys of women aged 15–49, longitudinal surveys of women who are pregnant or have recently given birth, and annual service delivery point surveys of health facilities (22). The Johns Hopkins School of Public Health received an online application through https://www.pmadata.org/data/available-datasets/request-accessdatasets, which was utilize to retrieve the PMA-Ethiopia datasets.

### Data quality control

The PMA Ethiopia fieldwork training started with a training of the entire field staff. The training started with a training of trainers (TOT) held from July 31st to August 3rd, 2019 in Addis Ababa. Throughout the training, all field staff were evaluated based on their performance on several written and phone-based assessments and class participation. All training participants were given in-depth instructions on survey protocols, the questionnaires, and guidance for conducting interviews using an Android phone. The resident enumerator training was conducted primarily in Amharic, whereas some small group sessions were conducted in Afan, Oromo, and Tigrigna (22). Data was extracted by using a checklist, cleaned, and recorded by using STATA software. Weighted values were used to ensure the representativeness of the sample data, weights through sample weight (FQweight) variable were calculated in each reproductive age group women PMA-ET.

### Data management and analysis

We conducted data analysis using SaTScan 9.6, ArcGIS 10.7, and STATA 14. Before conducting any statistical analysis, the data was weighted using the primary sampling unit, strata, and sampling weight (household sample weight) to ensure survey representativeness and instruct STATA to take the sampling design into account when calculating standard errors to produce accurate statistical estimates.

### Spatial autocorrelation analysis

Using Arc GIS 10.7 software, we evaluated whether home delivery in Ethiopia is dispersed, clustered, or randomly distributed using the spatial autocorrelation (Global Moran's I) statistic measure. If Moran's I value is zero, it indicates randomly distributed, close to +1 indicates clustered, and close to −1 shows dispersed home delivery (24).

### Getis-Ord Gi* hot spot and cold spot analysis

By calculating Gi* statistics for each area, Getis-Ord Gi* statistics were used to examine how spatial autocorrelation varies depending on the research location. For a *p*-value <0.05 at 95 CI, the *Z*-score was computed to verify the statistical significance of clustering. It is verified as a cold spot if the *Z*-score is less than −1.96 and as a hotspot area if it is larger than +1.96. Statistical output with high Gi* shows “hot spot” areas, whereas low Gi* means “cold spot” areas (25). The hot spot areas showed that there is a high proportion of home delivery and the cold spot showed that there is a low proportion of home delivery.

### Spatial interpolation

Using sampled clusters, unsampled areas' home delivery is predicted using the spatial interpolation technique (26). We employed the geostatistical ordinary Kriging spatial interpolation approach with ArcGIS 10.7 software to anticipate unsampled clusters. Home delivery is known for all enumeration areas in each PMAS survey year, yet it is also interesting to know about home delivery for other Ethiopian sites that were not selected.

### Spatial scan statistics

Using SaTScan version 9.6.1 software, the geographical locations of the statistically significant spatial window for home delivery were ascertained using a model spatial Kuldorff's Scan statistics based on Bernoulli (27). The Bernoulli model was applied by using Kuldorff's method for purely spatial analysis because the outcome variable has a Bernoulli distribution. According to the Bernoulli model, the women who deliver babies at home were referred to as the case and the women who deliver babies at a facility as the control group. This scanning window advances over the study area. Using 999 Monte Carlo replications' value of *p*-values and likelihood ratio tests, the most likely clusters were found by comparing them to the maximum spatial cluster size of less than 25% of the population. ArcGIS software version 10.7 was utilized to map the cluster and attribute of home delivery provided by SaTScan™, and we used non-overlapping choices by SaTScan version 9.6.1 to generate secondary clusters.

### Multilevel analysis

The PMA data had a hierarchical nature, this could violate the independence of observations and equal variance assumption of the traditional logistic regression model. This implies that there is a need to take into account the between-cluster variability by using advanced models. Therefore, a multilevel logistic regression model (both fixed and random effect) was fitted. Since the outcome variable was binary, standard logistic regression and multilevel logistic regression models were fitted. In the multilevel logistic regression model, we ran four models to estimate both fixed effects of the individual and community-level factors and random intercept of between-cluster variation. The first null or unconditional model contained no predictor variable used to decompose the amount of variance between cluster levels. The second model consisted of only individual-level factors, whereas the third model had only community-level variables. The final model controlled both individual and community factors (full model).

#### Intra-class correlation coefficient (ICC) and median odds ratio (MOR)

The Intra-Class Correlation (ICC) was used to express the random effects, or the amount of community variation, which are measures of variation in home delivery among communities or clusters. To determine whether there was variability or a clustering effect, the median odds ratio (MOR) was provided. When two clusters or EAs are randomly selected, it is defined as the median value of the odds ratio between the cluster with high odds of maternal home delivery and the cluster with lower odds of maternal home delivery (28).

The Likelihood Ratio (LR) test and deviance (−2LLR) values were utilized to evaluate model comparison and fitness since the models were nested (29). Accordingly, a mixed-effect logistic regression model (both fixed and random effect) was selected as the best-fitted model since it had the highest LLR and lowest deviance value. Variables with *p*-value <0.2 in the bi-variable analysis were considered in the multivariable logistic regression model. Adjusted Odds Ratios (AOR) with a 95% Confidence Interval (CI) and *p*-value <0.05 in the multilevel logistic model were used to declare significant factors associated with home delivery.

## Results

### Sociodemographic characteristics and distribution of home delivery among reproductive age group women in Ethiopia, PMA-ET 2019

Table 1 displays the absolute frequencies, percentages, and distributions of home deliveries among women in the reproductive age group according to various socio-demographic factors, along with associated data. The study comprised 5,796 participants in total, weighted for inclusion. 2,519 (43.46%) of the 5,796 women gave birth at home. Out of the study participants, over 2,493 (43.06%) women were between the 25–34 age range, with a mean age of 28.4 (±0.8) years. The majority of participants—3,658 (63.12%) were from rural areas and 4,991 (86.11%) were married. Home delivery was associated with women's age: 1,242 (54.67%) of women between the ages of 35 and 49 were more delivered at home than those between the ages of 15 and 24 (35.69%); rural inhabitants were more delivered at home than urban residents (386, 18.05%), with 2,133 (58.31%). Furthermore, there was a substantial correlation between wealth and home delivery: 542 women in the wealthiest class gave birth at home, but only 1,448 women (67.70%) in the lowest-income class did so. Education also showed a similar pattern: 688 (36.16%) of women with only a primary degree, 80 (11.63%) of women with a secondary degree, and 21 (4.69%) of women with a higher degree gave birth at home (Table 1).

**Table 1:** Sociodemographic characteristics and distribution of home delivery among reproductive age group women in Ethiopia, PMA-ET 2019 (*n* = 5,796).

Variables	Weighted frequency	Percent	Home delivery	
			No	Yes
			3,277 (56.54%)	2,519 (43.46%)
Age (in years)				
15–24	1,031	17.78%	663 (64.31%)	386 (35.69%)
25–34	2,493	43.06%	1,584 (63.54%)	909 (36.46%)
35–49	2,312	39.19%	1,030 (45.33%)	1,242 (54.67%)
Educational status				
No education	2,756	47.55%	1,026 (37.23%)	1,730 (62.77%)
Primary	1,904	32.85%	1,216 (63.87%)	688 (36.16%)
Secondary	688	11.87%	608 (88.77%)	80 (11.63%)
Higher	448	7.73%	427 (95.31%)	21 (4.69%)
Wealth index				
Poor	2,139	36.90%	691 (32.30%)	1,448 (67.70%)
Middle	1,027	17.71%	498 (48.49%)	529 (51.51%)
Rich	2,629	45.35%	2,087 (79.38%)	542 (20.62%)
Marital status				
Single	72	1.24%	55 (76.39%)	17 (23.61%)
Married	4,991	86.11%	2,843 (56.96%)	2,148 (43.04%)
Divorced	508	8.76%	291 (57.28%)	217 (42.72%)
Widowed	225	3.88%	88 (39.11%)	137 (60.89%)
Parity				
Prim parous	1,301	22.45%	977 (75.10%)	324 (24.90%)
Multi parous	4,385	75.66%	2,266 (51.68%)	2,119 (48.32%)
Grand-multi parous	110	1.90%	34 (30.91%)	76 (69.09%)
Community level literacy				
Low level	2,820	48.65%	1,095 (38.83%)	1,725 (61.17%)
High level	2,976	51.35%	2,182 (73.32%)	794 (26.68%)
Community level poverty				
Low level	3,039	52.43%	2,276 (74.89%)	763 (25.11%)
High level	2,757	47.57%	1,001 (36.31%)	1,756 (63.69%)
Residency				
Urban	2,138	36.88%	1,752 (81.95%)	386 (18.05%)
Rural	3,658	63.12%	1,525 (41.69%)	2,133 (58.31%)
Region				
Tigray	740	12.76%	537 (72.57%)	203 (27.43%)
Afar	309	5.33%	68 (22.01%)	241 (77.99%)
Amhara	1,060	18.28%	516 (48.68%)	544 (51.32%)
Oromia	1,173	20.23%	565 (48.17%)	608 (51.83%)
Somalia	143	2.46%	51 (35.66%)	92 (64.34%)
Benishangul	185	3.19%	99 (53.51%)	86 (46.49%)
SNNP	1,085	18.71%	558 (51.43%)	527 (48.57%)
Gambella	255	4.39%	173 (67.84%)	82 (32.16%)
Harari	224	3.86%	158 (70.54%)	66 (29.46%)
Addis Ababa	410	7.07%	380 (92.68%)	30 (7.32%)
Dire dawa	212	3.65%	172 (81.13%)	40 (18.87%)

### Prevalence of home delivery in Ethiopia

**Figure 3:**
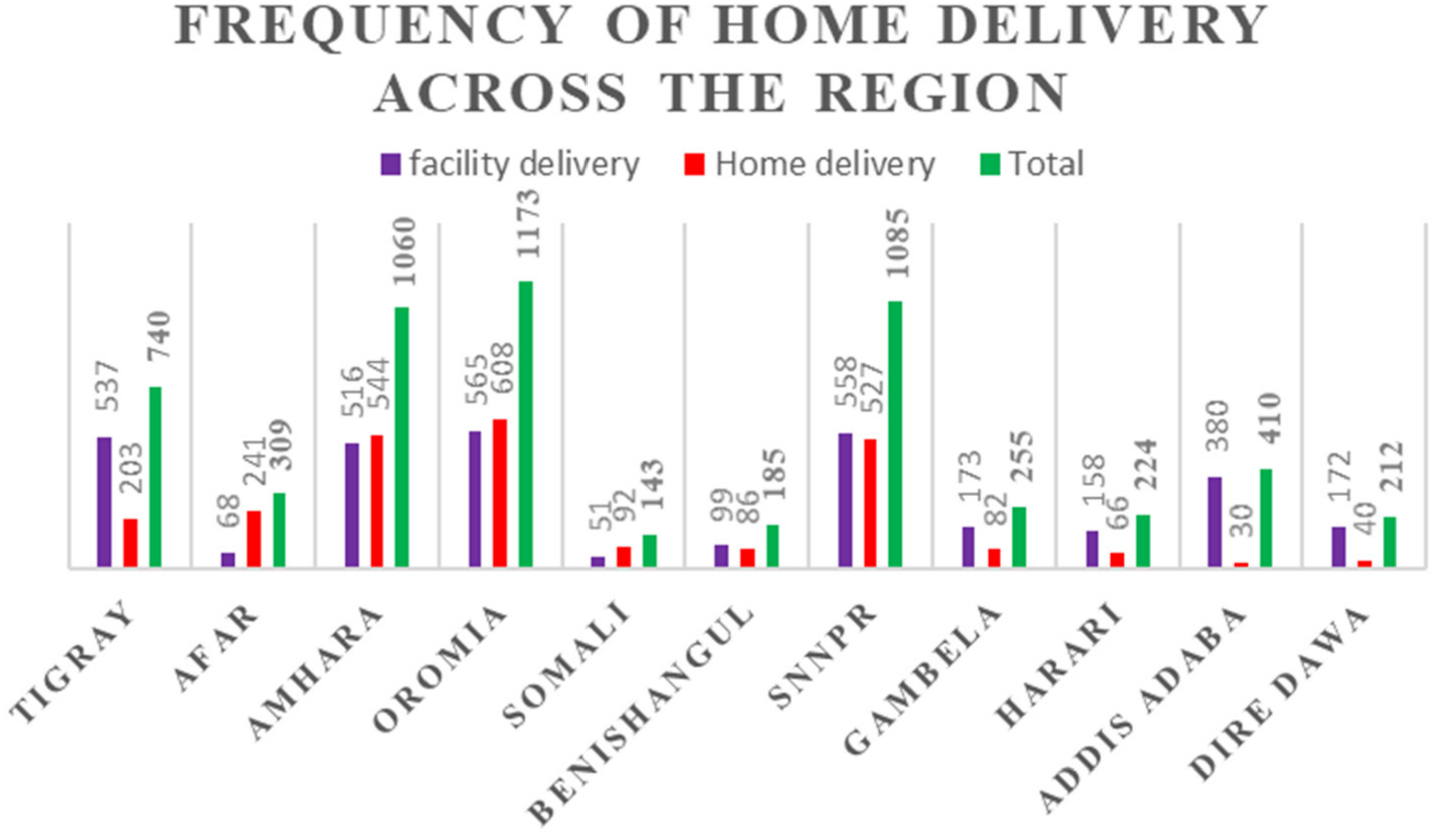
Frequency of home delivery among reproductive age women by administrative region in Ethiopia, PMA-ET 2019.

In Ethiopia, the prevalence of home delivery was 43.46% (95% CI: 42.18, 44.71). There were regional differences: home delivery was most common in the Afar, Amhara, Tigray, Oromia, Somalia, and SNNP regions, while it was least prevalent in Harari, Addis Abeba, and the Dire Dawa administrative zone of Ethiopia (Figure 3).

### Spatial autocorrelation (Global Moran's I) analysis

**Figure 4:**
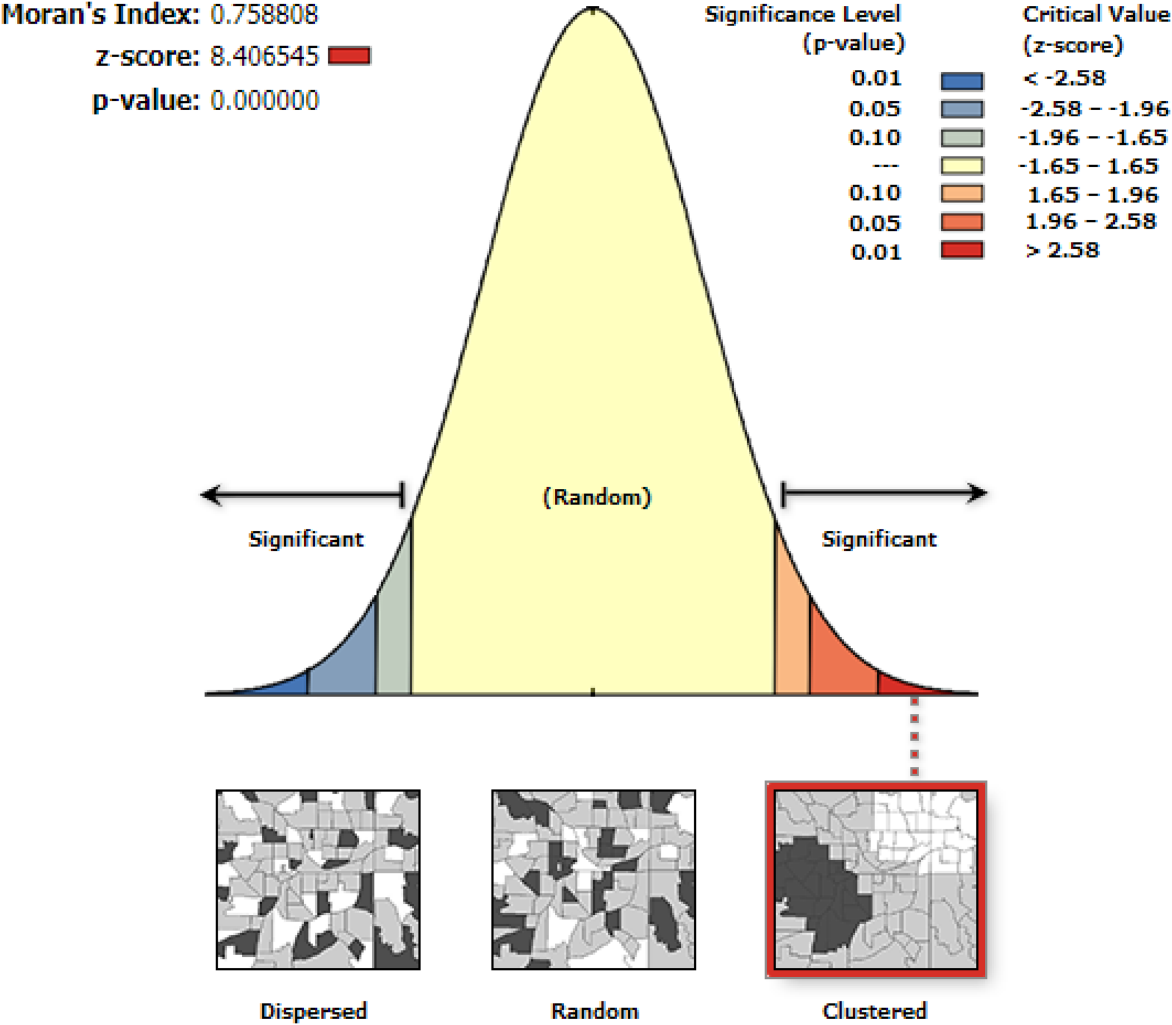
Spatial distribution of home delivery among reproductive age group women in Ethiopia, PMA-ET 2019.

Moran's I value was used to calculate the global spatial statistics. Figure 4 illustrates how the spatial variation in-home delivery among Ethiopian women of reproductive age was clustered with a *Z*-score of 8.40 and a Global Moran's I of 0.75 (*p*-value 0.0001), suggesting that the likelihood that this clustered pattern is the product of random chance is less than 1%.

### Getis-Ord Gi* hot spot and cold spot analysis

**Figure 5:**
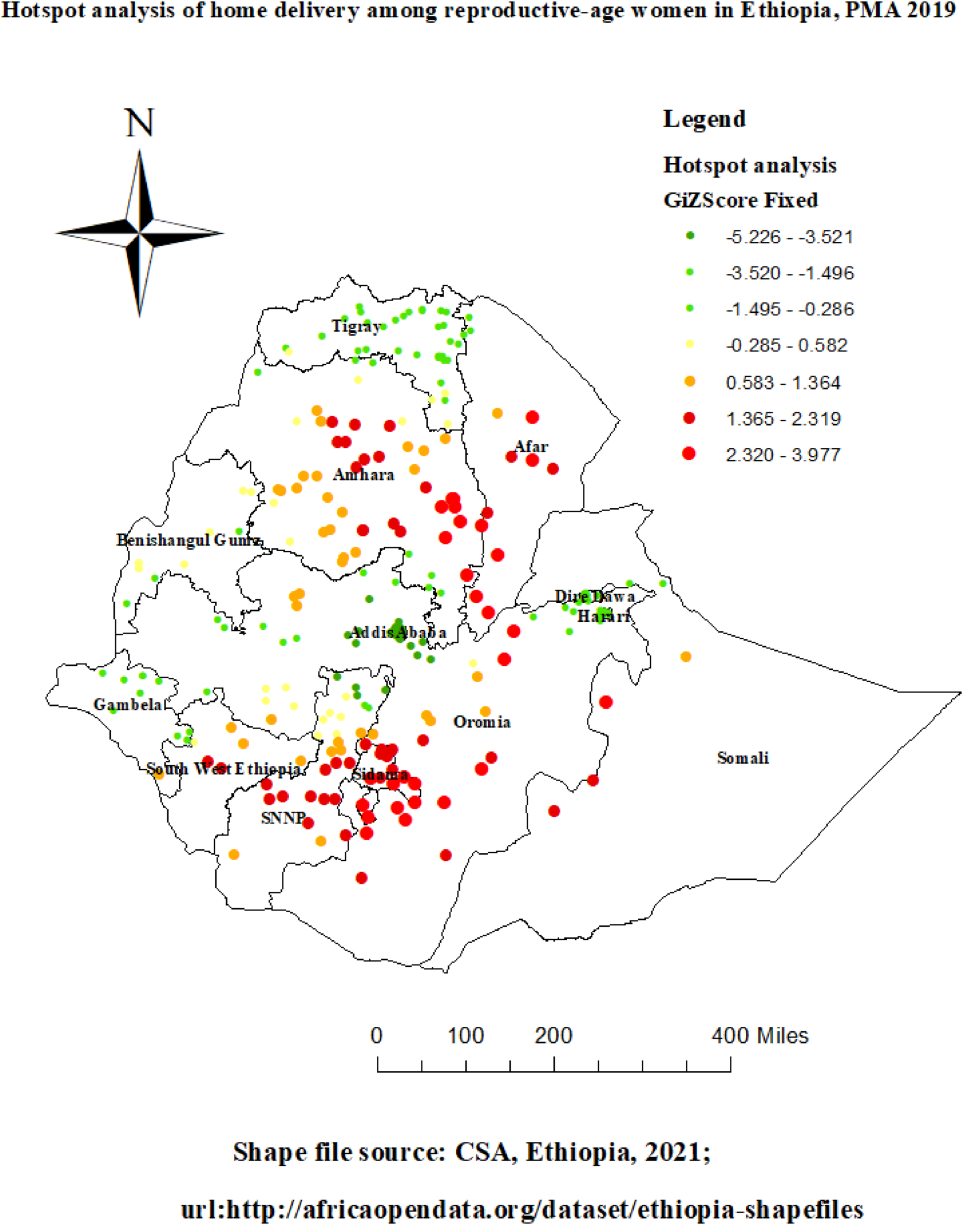
Hot spot analysis of home delivery among reproductive-age women in Ethiopia, PMA-ET 2019.

The women's hot and cold spots for home delivery were determined using the Getis-Ord Gi* analysis. A “hotspot” is indicated by a high Gi* statistical output, whereas a “cold spot” is indicated by a low Gi*. Red dots indicate high hot locations for home delivery, and green dots indicate low hot spots for home delivery at the 95 percent statistical significance level. Figure 5 illustrates the results of the spatial analysis at the cluster level. The results indicate that central and east Amhara region, South West Ethiopia, Somalia, Afar, Sidama, and Benishangul Gumize region of Ethiopia had statistically significant high hotspots for home delivery; on the other hand, most of Addis Ababa, Harare, Dire Dawa, Tigray, Gambella, and Benishangul Gumize region of Ethiopia had statistically significant low hotspots for home delivery.

### Spatial interpolation

**Figure 6:**
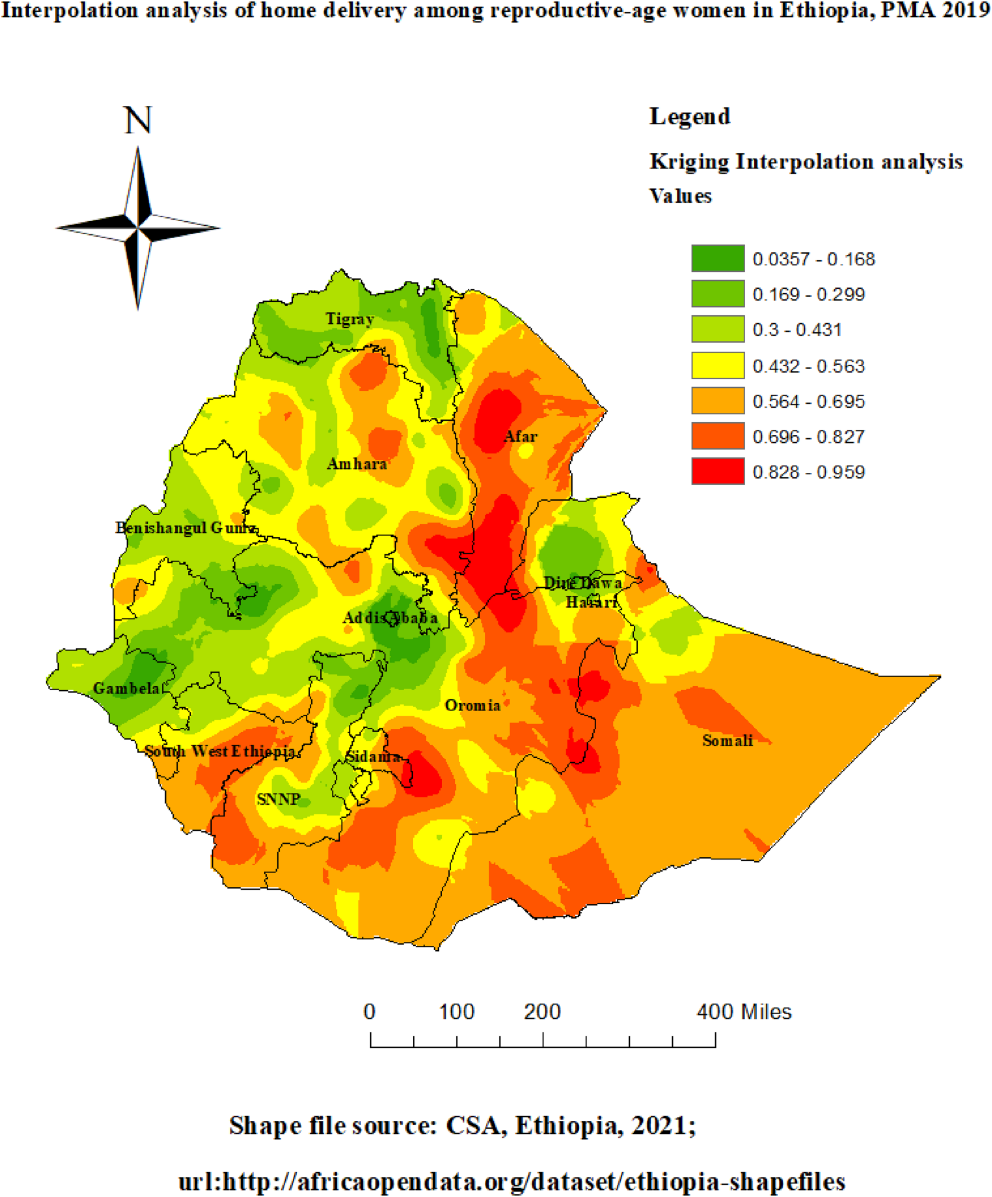
Interpolation of home delivery among reproductive-age women in Ethiopia, PMA-ET 2019.

In this study, home delivery in unobserved areas was predicted using the standard Kriging spatial interpolation technique. The expected home delivery increases from green to red-colored locations in PMA 2019 based on standard Kriging analysis. High-risk locations of anticipated home delivery were shown the red color, and low-risk zones were indicated the green colors. Figure 6 shows that compared to other regions, Afar, the majority of Oromia, Somalia, the Nation Nationality and People Region (SNNP), the majority of Amhara, Sidama, and the southwest region of Ethiopia were expected to be more dangerous for home delivery.

### Spatial scan statistics of home delivery

As shown in Table 2, primary and secondary significant clusters were detected using purely spatial cluster analysis that incorporated a total of 265 enumeration area ID (EA-ID). From the result of Kuldorff's Scan analysis, we identified 8(eight) spatial clusters, from which five of them were statistically significant *p*-value < 0.05.

**Table 2:** Kuldorff's scan statistics using the Bernoulli model summary.

Kuldorff's Scan statistics using the Bernoulli model summary	
Number of location (EA-ID)	265
Total number of population	5,829
Total number of case	2,544
Percent case in area	43.6

**Figure 7:**
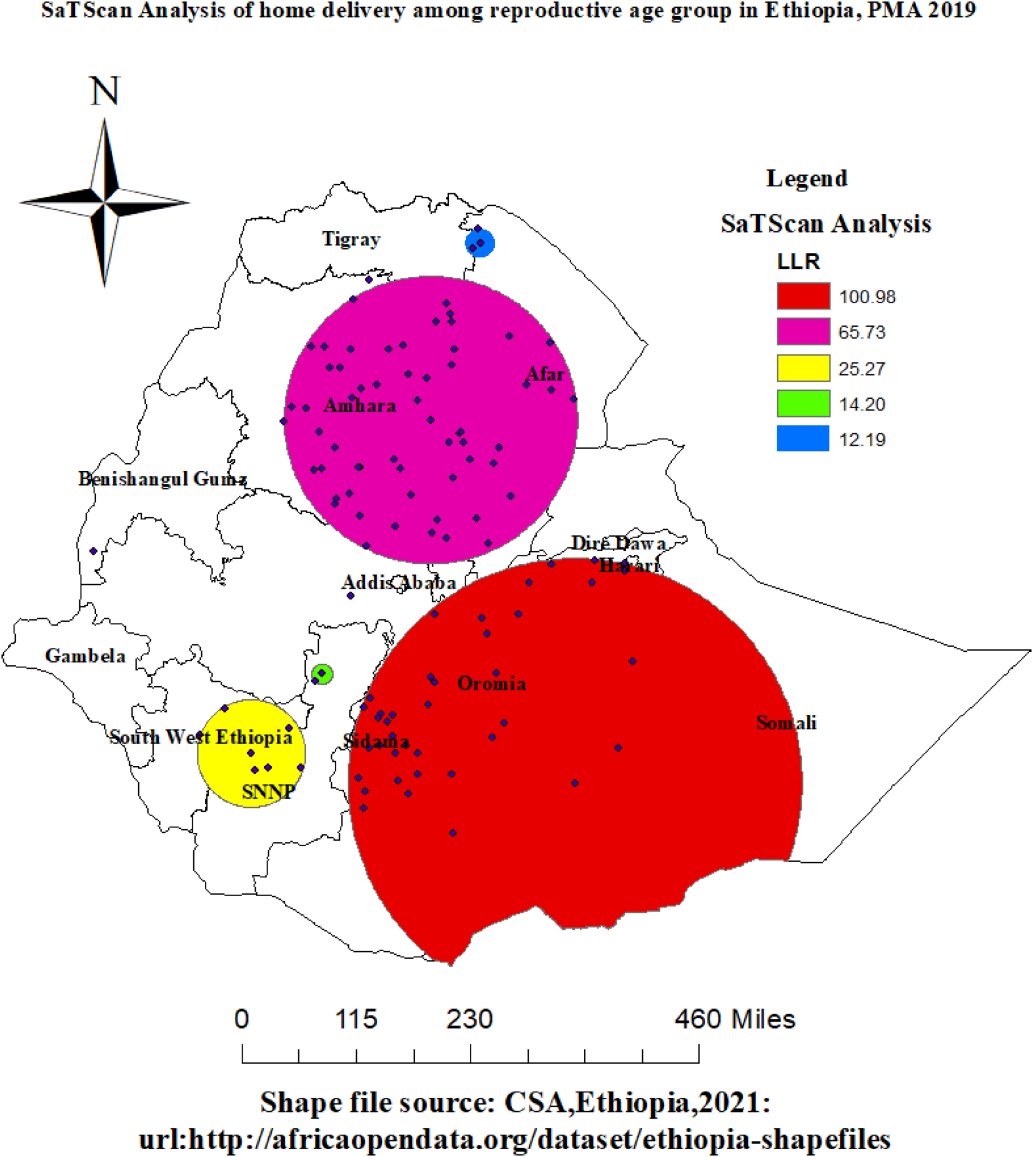
Spatial SaTScan analysis of home delivery among reproductive age group women in Ethiopia, PMA-ET 2019.

Its primary cluster, the red color ring spatial window, was typically located in the country's center, which includes Sidama, the majority of Somalia, Oromia, and a small portion of the Harari region. The center of this spatial window was 6.053746 N, 41.346590 E, with a radius of 365.77 km. The log-likelihood ratio (LLR) was 100.98, and the relative risk (RR: 1.64), at *p* < 0.001, was recorded. Figure 7 and Table 3 demonstrated that women who were inside the spatial window had a 1.64-fold increased likelihood of giving birth at home compared to those who were outside of it. Similarly, the Amhara and afar regions are encompassed by the peony pink hue spatial window, which has a radius of 233.20 km and a log-likelihood ratio (LLR) of 65.73 relative risk (RR: 1.45), at *p* < 0.001. The spatial window was centered at 11.362599 N, 39.242708 E. According to the data, women who were inside the spatial window were 1.45 times more likely to give birth at home than those who were outside of it. The yellow hue geographical window, which has a radius of 87.20 km and a log-likelihood ratio (LLR) of 25.27 relative risk (RR: 1.64), at *p* < 0.001, is centered at 6.498462 N, 36.622146 E. It includes parts of the SNNP region and southwest Ethiopia. According to the data, women who were inside the spatial window were 1.64 times more likely to give birth at home than those who were outside of it.

In addition, medium Apple color geographical window, which has a radius of 16.49 km and a log-likelihood ratio (LLR) of 14.20 relative risk (RR: 1.92), at *p* < 0.001, is centered at 7.657565 N, 37.657432 E. It includes smallest parts of the SNNP region. According to the data, women who were inside the spatial window were 1.92 times more likely to give birth at home than those who were outside of it. Northern regions of the afar region were covered by the Cretan Blue color spatial window, which has a radius of 23.48 km and a center position of 13.937987 N, 39.966918 E. The log-likelihood ratio (LLR) was 12.19 relative risk (RR: 1.67), with a *p*-value of less than 0.001. It was shown that the risk of home birth was 1.67 times higher for women who were inside the spatial window than for those who were outside of it. Refer to Table 3 and Figure 7.

**Table 3:** Significant clusters of home delivery among reproductive age women, PMAS 2019.

Type of cluster	# Cluster location	# Pop	# Case	RR	LLR	Coordinate/radius	*p*-value
Primary cluster	39	939	608	1.64	100.98	(6.053746 N, 41.346590 E)/365.77 km	<0.001
Secondary cluster 1	58	1,313	755	1.45	65.73	(11.362599 N, 39.242708 E)/233.20 km	<0.001
Secondary cluster 2	7	171	120	1.64	25.27	(6.498462 N, 36.622146 E)/87.20 km	<0.001
Secondary cluster 3	2	42	35	1.92	14.20	(7.657565 N, 37.657432 E)/16.49 km	<0.005
Secondary cluster 4	3	72	52	1.67	12.19	(13.937987 N, 39.966918 E)/23.48 km	<0.005

LLR, Log-Likelihood ratio; RR, Relative Risk.

### Multilevel analysis

#### Factor associated with home delivery in Ethiopia

##### The random effect analysis

Table 4 displays the random effect model. According to the null model, community-level factors account for approximately 41.53% of the total variation in home delivery. Different clusters had different home delivery rates, as indicated by the null model's highest MOR value (3.93). Additionally, the final model (Model 4) with the greatest PCV (64.61%) shows that both individual and community-level characteristics accounted for 64.61% of the variation in home delivery across neighborhoods. The best-fitting mode was identified as Model 4, which had the lowest deviation (5,968) and the highest Log likelihood (−2,984) among the models used to verify the fitness of the model.

**Table 4:** Model comparison and model fitness for multilevel logistic regression analysis.

Parameters	Null model	Model I	Model II	Model III
Random effect				
Community variance	2.32 [1.83, 2.94]	1.25 [0.96, 1.63]	0.88 [0.67, 1.14]	0.82 [0.63, 1.06]
ICC%	41.53%	27.6%	21.1%	20.0%
MOR	3.93 [3.50, 4.42]	2.88 [2.53, 3.29]	2.42 [2.06, 2.75]	2.33 [2.04, 2.65]
PCV%	1	46.12%	62.01%	64.61%
Model comparison				
AIC	6,750	6,546	6,115	6,022
BIC	6,763	6,646	6,208	6,201
LLR	−3,373	−3,258	−3,042	−2,984
Deviance	6,746	6,516	6,084	5,968

AIC, Akaike's information criterion; BIC, Bayesian information criterion; LLR, Log likelihood; MOR, Median Odd Ratio; ICC, Intra-class Correlation Coefficient and PCV (Proportional Change in Variance).

##### Fixed effect analysis

Model IV was used to evaluate the fixed effects, as indicated in Table 5 after it had been adjusted for individual and community-level factors. The individual and community-level factors were significant for home delivery in the multivariable mixed-effect binary logistic regression. Maternal education, wealth index, parity, age, and marital status, for example, were all significant individual-level predictors of home delivery. Place of residence, community poverty, and region were significant community-level factors influencing home delivery in Ethiopia.

**Table 5:** Multivariable multilevel logistic regression analysis of both individual and community-level factors associated with home delivery in Ethiopia.

Characteristics	Model I	Model II (95% CI AOR)	Model III (95% CI AOR)	Model IV (95% CI AOR)
Maternal age				
15–24		1		1
25–34		1.03 [0.83, 1.28]		1.11 [0.90, 1.38]
35–49		2.08 [1.63, 2.65]***		2.29 [1.80, 2.92]***
Marital status				
Married		1		1
Single		1.48 [0.73, 3.01]		1.75 [0.85, 3.62]
Divorced/separated		1.73 [1.33, 2.24]***		1.95 [1.50, 2.54]***
Widowed		2.72 [1.86, 3.96]***		2.96 [2.02, 4.34]***
Education status				
Higher education		1		1
No education		12.30 [6.79, 22.28]**		9.91 [5.44, 18.04]***
Primary education		7.59 [4.20, 13.71]**		6.62 [3.65, 12.00]***
Secondary education		3.13 [1.67, 5.85]**		2.99 [1.59, 5.63]***
Wealth status				
Rich		1		1
Poor		2.68 [2.14, 3.37]***		1.68 [1.31, 2.15]***
Middle		1.76 [1.40, 2.20]***		1.18 [0.93, 1.50]
Parity				
Prim parous		1		1
Multi parous		1.43 [1.16, 1.76]***		1.41 [1.14, 1.74]***
Grand-multiparous		1.70 [1.04, 2.80]		1.60 [0.98, 2.62]
Community level factors				
Place of residency				
Urban			1	1
Rural			3.06 [2.00, 4.69]*	2.05 [1.30, 3.22]***
Community level literacy				
High level			1	1
Low level			1.68 [1.17, 2.40]*	1.37 [0.94, 2.00]
Community level poverty				
Low level			1	1.00
High level			2.22 [1.48, 3.33]**	1.76 [1.14, 2.72]*
Region				
Tigray			1	1
Afar			5.44 [2.11, 14.01]*	6.59 [2.48, 17.49]***
Amhara			1.96 [1.17, 3.30]*	2.11 [1.22, 3.63]**
Oromia			2.59 [1.56, 4.31]***	3.48 [2.04, 5.92]***
Somali			6.95 [2.59, 18.66]**	7.12 [2.57, 19.71]***
Benishangul			2.74 [1.08, 6.95]*	2.67 [1.00, 7.07]*
SNNP			2.02 [1.19, 3.42]**	2.37 [1.36, 4.11]**
Gambella			1.83 [0.53, 6.32]	3.32 [0.86, 12.14]
Harari			1.01 [0.26, 3.85]	1.29 [0.32, 5.15]
Addis Ababa			0.64 [0.29, 1.43]	0.78 [0.34, 1.79]
Dire dawa			0.79 [0.21, 2.94]	0.89 [0.23, 3.48]

* (*P* < 0.05).

** (*P* < 0.01).

*** (*P* < 0.001).

Regarding individual-level factors, compared to women aged 15–24, women in the 35–49 age range had a 2.29 [AOR = 2.29, 95% CI: 1.80, 2.92] times higher likelihood of giving birth at home. Divorced or separated women had 1.95 times [AOR = 1.95, 95% CI: 1.50, 2.54] and widowed women had 2.96 times [AOR = 2.96, 95% CI: 2.02, 4.34] higher odds of becoming home delivery mothers than did married women. Regarding maternal educational status, mothers who had no education, primary education, and secondary education had 9.91 times [AOR = 9.91, 95% CI: 5.44, 18.04], 6.62 times [AOR = 6.62, 95% CI: 3.65, 12.00] and 2.99 times [AOR = 2.99, 95% CI: 1.59, 5.63] higher odds of giving birth at home compared to mothers who attained higher education, respectively. Women who had multiparous were 1.41 times [AOR = 1.41, 95% CI: 1.14, 1.74] more likely to deliver at home as compared to primiparous.

Women from the lowest wealth quintile were 1.68 times [AOR = 1.68; 95% CI: 1.31, 2.15] higher odds of giving birth at home as compared to their counterparts. In addition, community-level factors were significantly associated with home delivery, women who had high community-level poverty were 1.76 times [AOR = 1.76; 95% CI: 1.14, 2.72] higher odds of home delivery compared to women who had low community-level poverty. Regarding regions, women have higher odds of giving birth at home than in Tigray in Afar, Amhara, Oromia, Somali, Benishangul, and SNNP (6.59 [AOR = 6.59, 95% CI: 2.48, 17.49], 2.11 [AOR = 2.11, 95% CI: 1.22, 3.63], 3.48 [AOR = 3.48, 95% CI: 2.04, 5.92], 7.12 [AOR = 7.12, 95% CI: 2.57, 19.71], 2.67 [AOR = 2.67, 95% CI: 1.00, 7.07], and 2.37 [AOR = 2.37, 95% CI: 1.36, 4.11], respectively. For women residing in Gambella, Harrari, Addis Ababa, and Dire Dawa, there was not a significant difference in the place of delivery as compared to Tigray (Table 5).

## Discussion

According to the current study, the overall prevalence of home delivery in Ethiopia was 43.46% (95% CI: 42.18, 44.71). This finding is lower than the pocket study done in Kenya (67.3%) (21), in Ethiopia report from EDHS 2016 (73.44%) (18) and a pocket study done in Arbaminch zuria woreda (79.4%) (30), a study conducted in Anlemo District, southern Ethiopia (49.3%) (31), in Ghana (59%) (1). Whereas, this result is higher than the Arbaminch town Ethiopia (33.2%) (32). The discrepancy might be the study area, setting differences, cultural attitude toward health facility delivery, and infrastructure difference (access to the health facility and road) (9).

In this study, spatial analysis showed the presence of spatial heterogeneity using a significant positive spatial autocorrelation identified by Moran's I statistic as well as the significant local clusters identified by Getis-Ord Gi* analysis across the region. The spatial distribution of home delivery in Ethiopia was clustered (non-random), with higher levels of correlation in-home delivery rates between Ethiopian regions. The majority of Amhara, South West Ethiopia, Somalia, Afar, Sidama, and the Oromia area were shown to be statistically significant high hotspots for home delivery. Additionally, SaTScan analysis was used to evaluate the Getis-Ord analysis's hot spot region detection, and the results of the analysis provide confirmation. A prior study across Ethiopian areas revealed a considerable clustering of home delivery. The regions of Afar, Oromia, SNNP, and Somalia were determined to be hotspots for home delivery (9). The possible geographical variation of home delivery in the regions of Ethiopia might be sociodemographic factors, cultural behaviors to health facility delivery, and different infrastructures across the regions of Ethiopia.

In multilevel analysis, non-educated, primarily educated, and secondary educated women were more likely to deliver at home as compared to tertiary-educated women.This finding is supported by different studies conducted in Ethiopia (7, 9), in Tanzania (12). The possible reason could be education could influence women's overall empowerment enhancing their ability to have self-determination, access to information, and financial freedom to support themselves by taking transport to a health facility and pay for (if applicable) services, as well as to easily absorb health messages through the media and from health professionals (11). In addition, mothers living in rural areas were more likely to deliver at home. This finding collaborated with studies elsewhere (3, 4, 13). This result may be due to rural inhabitants usually having no or little access to maternal health and family planning programs as compared to urban residents (33). Furthermore, the reason for these might be due to the fact that in rural areas proportion of mothers with education is low, inaccessibility of services with long distances and transport, and mothers do not have better decision-making autonomy and better access to information than in urban mothers (13).

Maternal older age was significantly associated with the risk of home delivery in Ethiopia. This finding is supported by a study conducted in Bure district, East Guji, Ethiopia (34), a qualitative study conducted in south Ethiopia (35), and a study conducted in Kenya (36). The possible reason could be that younger mothers are more likely to give birth in contemporary medical facilities, whereas older mothers may rely on traditional methods. Additionally, the risk of giving birth at home was associated with women who were single or who had previously been married. This finding is supported studies conducted in Kenya (37), Nigeria (38), and Rwanda (39). The social stigmatism surrounding illegitimate births may be the reason for the low societal acceptance of unmarried status. Similarly, single women could feel less independent, alone, or unmotivated to seek medical care (40, 41).

In this study, women who had poor and high community-level poverty were more likely to give birth at home compared to their counterparts. This finding is supported by other studies done in Ethiopia (9, 10), Tanzania (12) and Kenya (21). The explanation for this could be that although government institutions in Ethiopia offer free expert delivery services, there could be related fees that only women from the wealthiest families can afford, both directly and indirectly. This could be due to community knowledge of health facility delivery and behavioral factors. In addition, women in low-income households might not be able to cover the family's expenses or the costs associated with giving birth, including transportation (4). Regarding region, mothers who live in Afar, Amhara, Oromia, SNNP, Benishangul, and Somali regional states of Ethiopia have highest odds of giving birth at home than in Tigray, which is consistent another study has been done in Ethiopia (9). This could be due to variations in infrastructure, social and cultural characteristics, and access to health services. Another reason could be that, in comparison to other areas, the maternal health facilities in the Tigray area are more readily available and easily accessible (11, 42). Identifying the high-risk areas of home delivery in regions of Ethiopia could be used to target intervention for home delivery reduction in high-risk areas. Therefore, identifying the spatial distribution and determinants of home delivery would help health planners and policymakers in Ethiopia.

### Strength and limitation of the study

The huge dataset from the PMA survey and the national representativeness of the study are its main advantages. To consider cluster correlations, multilevel multivariable analysis was employed. In addition to predicting unsampled/unmeasured portions of the nation, the spatiotemporal analysis was utilized to identify hotspot locations and most likely clusters. Nevertheless, this study's limitation is that the PMA 2019 data do not include information on media exposure, husband education, employment status, decision-making, perceived distance from the health facility, or other variables that may partially influence the delivery location. As with previous cross-sectional data, the PMA data do not support a causal connection. As such, these limitations need to be taken into account in any interpretation or conclusion drawn from this work.

## Conclusions

Ethiopia has a high home childbirth prevalence. The results of this study can be used to the spatial variation in home birth among Ethiopian women in the reproductive age group. Home delivery was statistically found to be a significantly high hot spot in Somalia, Afar, Sidama, most of the South Nation Nationality and People area (SNNP), most of Amhara, southwest Ethiopia, and the Oromia region of Ethiopia. Significant factors that predicted home delivery in Ethiopia were women with lower levels of education, poor wealth, living in rural areas, high levels of community poverty, divorced or separated widowed marital status, and older maternal ages. To alleviate home delivery, spatiotemporal clusters around the nation allow for timely spatial targeting considerations. Therefore, to create an efficient intervention program to decrease home delivery, national and regional authorities, as well as health planners, should prioritize the identified hot spot clusters.

## Data Availability

Publicly available datasets were analyzed in this study. This data can be found here: https://www.pmadata.org/data/available-datasets/request-accessdataset.

## References

[1] Ganle JK Mahama MS Maya E Manu A Torpey K Adanu R. Understanding factors influencing home delivery in the context of user-fee abolition in Northern Ghana: evidence from 2014 DHS. Int J Health Plann Manage. (2019) 34(2):727–43. 10.1002/hpm.2731 30657200

[2] Smith SL Shiffman J. Setting the global health agenda: the influence of advocates and ideas on political priority for maternal and newborn survival. Soc Sci Med. (2016) 166:86–93. 10.1016/j.socscimed.2016.08.013 27543685 PMC5034850

[3] Nigatu AM Gelaye KA Degefie DT Birhanu AY. Spatial variations of women’s home delivery after antenatal care visits at lay Gayint District, Northwest Ethiopia. BMC Public Health. (2019) 19(1):1–14. 10.1186/s12889-019-7050-4 31159775 PMC6545631

[4] Chernet AG Dumga KT Cherie KT. Home delivery practices and associated factors in Ethiopia. J Reprod Infertil. (2019) 20(2):102. 31058055 PMC6486567

[5] Devkota B Maskey J Pandey AR Karki D Godwin P Gartoulla P Determinants of home delivery in Nepal–A disaggregated analysis of marginalised and non-marginalised women from the 2016 Nepal demographic and health survey. PLoS One. (2020) 15(1):e0228440. 10.1371/journal.pone.0228440 31999784 PMC6992204

[6] Bang RA Bang AT Reddy MH Deshmukh MD Baitule SB Filippi V. Maternal morbidity during labour and the puerperium in rural homes and the need for medical attention: a prospective observational study in Gadchiroli, India. BJOG. (2004) 111(3):231–8. 10.1111/j.1471-0528.2004.00063.x 14961884

[7] Kasaye HK Endale ZM Gudayu TW Desta MS. Home delivery among antenatal care booked women in their last pregnancy and associated factors: community-based cross sectional study in Debremarkos town, North West Ethiopia, January 2016. BMC Pregnancy Childbirth. (2017) 17(1):1–12. 10.1186/s12884-017-1409-2 28705188 PMC5512956

[8] Hagos S Shaweno D Assegid M Mekonnen A Afework MF Ahmed S. Utilization of institutional delivery service at Wukro and Butajera districts in the Northern and South Central Ethiopia. BMC Pregnancy Childbirth. (2014) 14(1):1–11. 10.1186/1471-2393-14-178 24886375 PMC4047000

[9] Tessema ZT Tiruneh SA. Spatio-temporal distribution and associated factors of home delivery in Ethiopia. Further multilevel and spatial analysis of Ethiopian demographic and health surveys 2005–2016. BMC Pregnancy Childbirth. (2020) 20:1–16. 10.1186/s12884-019-2665-0 PMC7268646 32493302

[10] Tiruneh SA Lakew AM Yigizaw ST Sisay MM Tessema ZT. Trends and determinants of home delivery in Ethiopia: further multivariate decomposition analysis of 2005–2016 Ethiopian Demographic Health Surveys. BMJ Open. (2020) 10(9):e034786. 10.1136/bmjopen-2019-034786 32873665 PMC7467527

[11] Yebyo H Alemayehu M Kahsay A. Why do women deliver at home? Multilevel modeling of Ethiopian National Demographic and Health Survey data. PLoS One. (2015) 10(4):e0124718. 10.1371/journal.pone.0124718 25874886 PMC4398378

[12] Mrisho M Schellenberg JA Mushi AK Obrist B Mshinda H Tanner M Factors affecting home delivery in rural Tanzania. Trop Med Int Health. (2007) 12(7):862–72. 10.1111/j.1365-3156.2007.01855.x 17596254

[13] Abdella M Abraha A Gebre A Reddy PS. Magnitude and associated factors for home delivery among women who gave birth in last 12 months in Ayssaita, Afar, Ethiopia-2016. A community based cross sectional study. Glob J Fertil Res. (2017) 2(1):030–9. 10.17352/gjfr.000009

[14] Ramezani Siakhulake F Tabatabaei SM Mohammadi M Behmanesh Pour F. Home delivery practices among pregnant women in southeast of Iran and associated factors after the implementation of the health transformation plan: a case-control study. Women’s Health Bull. (2021) 8(3):142–51. 10.30476/whb.2021.91277.1122

[15] Abubakar S Adamu D Hamza R Galadima JB. Determinants of home delivery among women attending antenatal care in Bagwai town, Kano Nigeria. Afr J Reprod Health. (2017) 21(4):73–9. 10.29063/ajrh2017/v21i4.8 29624953

[16] Scott NA Henry EG Kaiser JL Mataka K Rockers PC Fong RM Factors affecting home delivery among women living in remote areas of rural Zambia: a cross-sectional, mixed-methods analysis. Int J Womens Health. (2018) 10:589. 10.2147/IJWH.S169067 30349403 PMC6181475

[17] Idris S Gwarzo U Shehu A. Determinants of place of delivery among women in a semi-urban settlement in Zaria, Northern Nigeria. Ann Afr Med. (2006) 5(2):68–72.

[18] Central Statistical Agency (CSA) [Ethiopia] and ICF. Ethiopia Demographic and Health Survey Final Report. Addis Ababa: Central Statistical Agency (2016).

[19] Simfukwe ME. Factors contributing to home delivery in Kongwa District, Dodoma-September 2008. Dar Es Salaam Med Stud J. (2011) 18(1):13–22. 10.4314/dmsj.v18i1.71001

[20] Abebe F Berhane Y Girma B. Factors associated with home delivery in bahirdar, Ethiopia: a case control study. BMC Res Notes. (2012) 5(1):1–6. 10.1186/1756-0500-5-1 23176369 PMC3554461

[21] Ogolla JO. Factors associated with home delivery in West Pokot County of Kenya. Adv Public Health. (2015) 2015:1–6. 10.1155/2015/493184

[22] Zimmerman L Desta S Yihdego M Rogers A Amogne A Karp C Protocol for PMA-Ethiopia: a new data source for cross-sectional and longitudinal data of reproductive, maternal, and newborn health. Gates Open Res. (2020) 4:1–16. 10.12688/gatesopenres.13161.1 PMC7593701 33150302

[23] Worldometers. Ethiopia Population (LIVE). (2024). Available from: https://www.worldometers.info/world-population/ethiopia-population/?fbclid=IwAR03wyazDw5aYikmxQTaamOAKIum3B0DzYk4St21M8A9tT3ESGtTv6kyN0U (Cited November 18, 2024).

[24] Tsai P-J Lin M-L Chu C-M Perng C-H. Spatial autocorrelation analysis of health care hotspots in Taiwan in 2006. BMC Public Health. (2009) 9(1):1–13. 10.1186/1471-2458-9-464 20003460 PMC2799414

[25] Wulder M Boots B. Local spatial autocorrelation characteristics of remotely sensed imagery assessed with the Getis statistic. Int J Remote Sens. (1998) 19(11):2223–31. 10.1080/014311698214983

[26] Krivoruchko K. Empirical Bayesian Kriging. California: USA: ESRI: Redlands, CA (2012). Available online at: http://www.esri.com/news/arcuser

[27] Kulldorff M. A spatial scan statistic. Commun Stat Theory Methods. (1997) 26(6):1481–96. 10.1080/03610929708831995

[28] Larsen K Merlo J. Appropriate assessment of neighborhood effects on individual health: integrating random and fixed effects in multilevel logistic regression. Am J Epidemiol. (2005) 161(1):81–8. 10.1093/aje/kwi017 15615918

[29] Snijders TA Bosker RJ. Multilevel Analysis: An Introduction to Basic and Advanced Multilevel Modeling. Thousand Oaks, CA: Sage (2011).

[30] Ayele G Tilahune M Merdikyos B Animaw W Taye W. Prevalence and associated factors of home delivery in Arbaminch Zuria district, Southern Ethiopia: community based cross sectional study. Science. (2015) 3(1):6–9. 10.11648/j.sjph.20150301.12

[31] Ibrahim S Analyzing prevalence of home delivery and associated factors in Anlemo District, Southern Ethiopia. Int Ann Med. (2017) 1(6):1–8. 10.24087/IAM.2017.1.6.169

[32] Firdawek E Aragu D. Magnitude and determinants of antenatal and delivery service utilization in Arba Minch town, South Ethiopia. Sci J Public Health. (2015) 3(3):339. 10.11648/j.sjph.20150303.16

[33] Woldemicael G. Recent fertility decline in Eritrea: Is it a conflict-led transition? Demogr Res. (2008) 18:27–58. 10.4054/DemRes.2008.18.2

[34] Teressa B Legesse E Nigussie T Deriba BS Guye AH Girma D Determinants of home delivery among reproductive age women in Bore District, East Guji Zone, Ethiopia: a case–control study. Front Global Women’s Health. (2024) 5:1236758. 10.3389/fgwh.2024.1236758 PMC11190296 38912412

[35] Temesgen T Figa Z. Why do pregnant mothers prefer to give birth at home after they attended antenatal care visits in Southern Ethiopia? A phenomenological study design. Curr Women’s Health Rev. (2024) 20(1):62–8. 10.2174/1573404819666230120122906

[36] Moindi RO Ngari MM Nyambati VC Mbakaya C. Why mothers still deliver at home: understanding factors associated with home deliveries and cultural practices in rural coastal Kenya, a cross-section study. BMC Public Health. (2015) 16:1–8. 10.1186/s12889-016-2780-z PMC4738797 26842657

[37] Ono M Matsuyama A Karama M Honda S. Association between social support and place of delivery: a cross-sectional study in Kericho, Western Kenya. BMC Pregnancy Childbirth. (2013) 13:1–9. 10.1186/1471-2393-13-214 24261639 PMC4222494

[38] Nduka I Nduka E. Determinants of noninstitutional deliveries in an Urban Community in Nigeria. J Med Invest Practice. (2014) 9(3):102. 10.4103/9783-1230.144770

[39] Regassa LD Tola A Weldesenbet AB Tusa BS. Prevalence and associated factors of home delivery in Eastern Africa: further analysis of data from the recent Demographic and Health Survey data. SAGE Open Med. (2022) 10:20503121221088083. 10.1177/20503121221088083 35342629 PMC8949735

[40] Ahinkorah BO. Non-utilization of health facility delivery and its correlates among childbearing women: a cross-sectional analysis of the 2018 Guinea demographic and health survey data. BMC Health Serv Res. (2020) 20:1–10. 10.1186/s12913-020-05893-0 PMC7650152 33167985

[41] Nyblade L Stockton MA Giger K Bond V Ekstrand ML Lean RM Stigma in health facilities: why it matters and how we can change it. BMC Med. (2019) 17:1–15. 10.1186/s12916-019-1256-2 30764806 PMC6376713

[42] Alemi Kebede KH Teklehaymanot AN. Factors associated with institutional delivery service utilization in Ethiopia. Int J Women’s Health. (2016) 8:463. 10.2147/IJWH.S109498 27672342 PMC5026219

